# Informed consent and bioethical advances in clinical settings

**DOI:** 10.3389/fpsyg.2025.1654586

**Published:** 2025-09-08

**Authors:** Maria Laura Giacobello

**Affiliations:** Department of Ancient and modern civilizations, University of Messina, Messina, Italy

**Keywords:** bioethics, doctor-patient relationship, adherence, psychological well-being, artificial intelligence, informed consent, explainability

## Abstract

The increasing integration of artificial intelligence (AI) in healthcare, despite the still uncertain implications for clinical practice, underscores the vast array of opportunities it brings to medicine. The benefits of technological enhancement in this domain are clear and substantial. However, this same context gives rise to equally significant ethical concerns, particularly in relation to data security, confidentiality, equitable access, and the attribution of responsibility. AI’s emergence in clinical settings introduces complexities that traditional informed consent procedures are not fully equipped to address, prompting ethical, legal, and practical concerns around information delivery and patient autonomy. Effective physician-patient communication is critical to ensuring informed and voluntary adherence to treatment. Such communication also plays a pivotal role in supporting patients’ psychological well-being and encouraging their active involvement in care. AI’s role as a third party in the therapeutic relationship necessitates a serious examination of the new risks it introduces. Bioethics must provide a prudent and critical framework to evaluate and ethically guide the development and deployment of such technologies. This constitutes both a technical and moral challenge. In Ethics of Artificial Intelligence, Floridi observes that major ethical frameworks for AI converge with the principles first formulated by Beauchamp and Childress in Principles of Biomedical Ethics (1979): autonomy, non-maleficence, beneficence, and justice. Floridi argues for the inclusion of a fifth principle, explicability, as essential in addressing the opacity of AI systems. Explicability, requiring that AI processes be comprehensible and transparent, is intrinsically linked to the principle of autonomy and its practical expression: informed consent. The integration of AI into clinical practice directly affects the moment when a physician’s duty to inform meets the patient’s right to autonomy. The traditional principlist model identifies challenges in the communication of information: an area now further complicated by AI’s opacity. This raises pressing questions about the physician’s obligation to disclose AI involvement in care decisions. Ultimately, as the therapeutic relationship evolves from a dyadic to a triadic model, physician, patient, and AI, there is a need to reassess informed consent practices, with sustained commitment to the core ethical values of transparency and autonomy.

## 1 Introduction. Artificial intelligence: a third party between patient and doctor

The increasing integration of artificial intelligence (AI) into healthcare (Aung et al., 2021), despite the overall impact on clinical practice still being uncertain (Mittelstadt, 2021), highlights the wide range of possibilities emerging in medicine as it becomes more widely adopted: greater accuracy in diagnosis, pathophysiology, therapy, and prognosis; the ability to quickly consult the most up-to-date guidelines; improvements in particularly critical areas such as triage prioritization and emergency care management; assistance in clinical trials; contributions to precision medicine; and robotics, both in surgical contexts and in the care of the elderly and people with disabilities. The benefits stemming from the advancement of new technologies in healthcare are, therefore, both evident and numerous (Charlotte and Drazen, 2023). AI, in particular, has the potential to significantly assist healthcare professionals, and it is broadly hoped that its contribution will continue to grow, especially in managing repetitive, administrative, or high-risk tasks. Such support may even allow physicians to redirect their time and energy toward strengthening the patient-physician relationship (CNB - CNBBVS, 2020, p. 3; pp. 8–9).

Nonetheless, alongside these promising developments, equally pressing ethical challenges are emerging (Weiner et al., 2025). Among the most critical concerns are those related to data security, patient confidentiality, the fair use of information and equitable access to AI-driven tools and systems. It is important not to overlook the clear ethical implications tied to the principle of justice: the use of AI in medicine, still largely concentrated in high-income countries and among patients with higher socioeconomic status, risks further exacerbating the already significant disparities in access to specialized healthcare among different segments of the population. Moreover, the question of how responsibility and accountability should be assigned in AI-assisted clinical decisions remains a key issue (Scaffardi, 2022; Cestonaro et al., 2023).

The advent of artificial intelligence in healthcare also introduces, from another perspective explicitly considered here, additional layers of complexity that cannot be adequately addressed through the traditional frameworks of informed consent. This raises a range of ethical, legal, and practical concerns regarding the patient’s right to information and personal autonomy. Informed consent is, in fact, the cornerstone of all ethical medical practice (Paterick et al., 2008, 2020), as it ensures that patients understand the procedures they undergo, the associated risks, and the available alternatives.

Effective communication between physician and patient, grounded in the ethical standards of informed consent, is thus an essential prerequisite for achieving full and conscious adherence to prescribed treatments. Such communication directly influences how patients perceive the proposed interventions and their willingness to comply with therapeutic recommendations. Therapeutic compliance, in turn, plays a crucial role in the patient’s psychological well-being, fostering a sense of active participation in the care relationship (Guerra, 2021).

It is, therefore, imperative to give serious consideration to the risks that arise when AI is inserted as a third party in the doctor-patient relationship (Gensabella Furnari, 2005)^1^. These risks demand the attention of bioethics, which is called upon to provide a critical, balanced, and prudent evaluation, capable of ethically steering the advancement and application of new technological systems in healthcare (Palazzani, 2020).

The first and perhaps most pervasive risk is that AI, rather than remaining a supportive tool, may become intrusive, ultimately replacing the physician in certain contexts and tasks. It is, however, essential that AI be maintained as an instrument subordinate to the clinician’s judgment. The competence, professional autonomy, and responsibility of healthcare providers must not, and cannot, be replaced by technology.

If the aim is to promote a model of medicine that is truly patient-centered, it is crucial to address the serious danger of undermining the therapeutic relationship (Teasdale et al., 2024), which should remain the axis around which all healthcare practice revolves. This is also reflected in the recent surge of interest in care ethics (Adorno, 2019).

Medicine is, at its core, a relational practice. The communication between doctor and patient is not merely a technical step for delivering information and obtaining consent (Cocanour, 2017), nor a neutral process of data exchange; it constitutes a fundamental part of the “time of care.”^2^ To disregard the value of communication in the clinical encounter is to erode the ethical foundation of the therapeutic relationship.

This underscores the need for a critical assessment of the new opportunities brought by AI specifically within the healthcare domain, in order to promote a medicine with machines rather than a medicine of machines. As stated in the joint opinion of the Italian Committee for Bioethics and the Italian Committee for Biosafety, Biotechnology and Sciences of Life. Artificial Intelligence and Medicine: Ethical Aspects (29 May 2020): «The goal is to identify the ethical conditions for a development of AI that does not forsake certain aspects of our humanity, in a new “digital humanism”, for medicine “with” machines and not “of” machines. In the awareness that it is man who builds the technology and that technology is not a neutral tool, as it inevitably changes the doctor-patient relationship itself» (CNB - CNBBVS, 2020, p. 9).

It is likely that physicians who adopt AI will replace those who do not, but AI itself will not replace physicians, even if its use may offer advantages in certain areas.

## 2 The importance of explainability for effective human-machine collaboration in medicine

From its inception, bioethics has been engaged in rethinking the patient-physician relationship, with particular attention to the transformations brought about by the widespread use of modern technology and its derivatives. Today, the integration of AI significantly expands the role of the “third party” that technology has long occupied within this relationship, marking a further qualitative leap beyond modern techniques (Heuvel van den et al., 2025).

Whereas modern technology could no longer be viewed as a mere tool to be used or set aside at will (Heidegger, 1976), this issue becomes even more pronounced with AI, which represents its most recent and powerful expression: digital technology today truly becomes fully integrated environment, a space for interaction (Pessina, 2023; Valera, 2022). Therefore, envisioning a sustainable coexistence between life and technology undoubtedly also requires strengthening philosophical and anthropological studies (Bertolaso and Marcos, 2023).

Current evidence increasingly suggests the urgency of rethinking the patient-physician relationship paradigm to consciously include AI as a third actor within the therapeutic alliance (Borghi et al., 2025).

Although the remarkable complexity and effectiveness of AI systems across various professional domains often leads to their association with the notion of natural intelligence, they are, in fact, a specific manifestation of digital technology. AI should be understood as a complex network of computational mechanisms, a phenomenon of automation that encompasses capabilities such as classification, evaluation, identification, planning, and prediction. However, it excludes any meaningful reference to concepts such as identity, consciousness, or autonomy (Faggin, 2022; Mitchell, 2019).

Algorithms, in any case, give new substance to the ambition of a medicine that is as objective as possible, less exposed to the variables of the physician’s inevitably subjective assessment, and able to offer greater guarantees through more reliable prognoses, made possible by access to and management of data volumes that were previously unimaginable. However, if algorithms «are considered “reliable and neutral” in themselves, only for the fact that their methods are represented through measurable, mathematical systems» (CNB - CNBBVS, 2020, p. 10), it is then necessary to remember that, on closer inspection, they process data collected and selected by human beings and are themselves constructed by human beings. It is clear, therefore, that since artificial intelligence conveys an action program always imprinted by humans (Floridi and Cabitza, 2021), it inevitably reflects the biases of those who design it: «The discrimination does not come from the machine but from man who selects the data and develops the algorithms» (CNB - CNBBVS, 2020, p. 11).

Thus, data collected by selecting a particular group of patients, while excluding others, may yield results that are far removed from the ideals of precision medicine. This selective approach can lead to erroneous clinical evaluations of “that” specific patient whom the physician is called upon to assess in each unique context. Moreover, regardless of the degree of accuracy attained, programmers themselves are often unable to explain the reasoning process followed by the machine in reaching a given decision, due to the inherent lack of transparency associated with automation. This is the well-known “black box” problem, which underscores the opacity of AI systems (Director, 2025).

In practice, it becomes impossible to interpret the immense volume of calculations performed by the algorithm in order to fully understand how the machine arrived at its decision. As Ben Mittelstadt notes in his report The Impact of Artificial Intelligence on the Doctor-Patient Relationship, commissioned by the Steering Committee for Human Rights in the Fields of Biomedicine and Health (CDBIO) of the Council of Europe:

«In cases where AI systems provide some form of clinical expertise, for example by recommending a particular diagnosis or interpreting scans, this requirement to explain one’s decision-making would seemingly be transferred from doctor to AI system, or at least to manufacturer of AI system. The difficulty of explaining how AI systems turn inputs into outputs poses a fundamental challenge for informed consent. Aside from the patient’s capacity to understand the functionality of AI systems, in many cases patients simply do not have sufficient levels awareness to make free and informed consent possible. AI systems use unprecedented volumes of data to make their decisions, and interpret these data using complex statistical techniques, both of which increase the difficulty and effort required to remain aware of the full scope of data processing and clinical analysis informing one’s diagnosis and treatment» (Mittelstadt, 2021, p. 5).

Truly, AI’s ability to record data and combine them makes it increasingly indispensable in a growing and potentially unlimited number of tasks, with the consequence that in a fully digitized environment, at times data are readable exclusively by machines.

A legitimate question arises, then, as to whether it can be considered a responsible choice to entrust exclusively to algorithms the prognosis of patients, thus directing, on the basis of the predictions they return to us, decisions concerning health, and at times even life itself: for example, the choice of whether to continue or suspend treatment, or even that of priorities in life-saving care in cases of shortage of medical resources should not be taken conditioned by extra-health considerations, nor should they be affected by discriminatory and stigmatizing orientations.

In any case, since the risk of discrimination stems from humans inputting data and designing algorithms on the basis of selections that are not inclusive and influenced by bias, it is imperative that the use of AI, in general, but more than ever in medicine, be supported by an ethical orientation and a responsible attitude of constant vigilance (Jeyaraman et al., 2023), which intervenes from the design of the machines, to also cross the stages of analysis and validation of the results achieved.

Technological tools are never ethically neutral, as their very design is oriented toward a purpose. In this respect, the development of any technology must be understood as a moral act, one that inherently entails human responsibility (Floridi, 2023; Giacobello, 2019).

Respecting this premise, namely, the ethical configuration of AI through careful data selection for training and appropriate algorithm choice, is not, however, sufficient to ensure the equitable use of AI in medicine. It is essential to remember that its role should remain that of a support tool for clinical judgment. Indeed, there is a tangible risk that the distinctive competencies at the core of medical practice may be progressively eroded if clinicians place excessive trust in AI, thereby neglecting the unique clinical and existential reality of the individual patient under their care.

It is therefore essential that physicians resist the temptation to delegate to AI the responsibility of predicting, and thus deciding, the fate of the patients with whom they engage. Rather, they must remain the privileged interpreters of scientific data and the final decision-makers, playing a central role, together with the patient, in a caring relationship grounded in professionalism, empathy, and trust (Sung, 2023).

From this role and responsibility, the physician must be prepared to confront and share with the patient the inherent risks involved in the use of such new tools; chief among them, the issue of opacity, which complicates the physician’s ability to serve as the ultimate interpreter of the AI-generated outcomes. In practical terms, it is virtually impossible to critically scrutinize and explain to the patient the rationale behind decisions influenced by a machine whose logic remains fundamentally inaccessible.

This circumstance, clearly, «raises problems for the doctor in relation to the machine (whether or not to rely on the algorithms) and in relation to the patient, to whom the doctor cannot provide an explanation and transparent information» (CNB - CNBBVS, 2020, p. 11).

A challenge, both technical and ethical, takes shape here.

In Ethics of Artificial Intelligence, Floridi (2022), after analyzing what he considers the most significant documents concerning ethical principles for AI, identifies a clear alignment with the core principles first formulated in 1979 by Beauchamp and Childress in their seminal work Principles of Biomedical Ethics, which are: autonomy, non-maleficence, beneficence, and justice.

To these four principles, however, Floridi considers it essential to add a fifth, particularly crucial in the context of AI, to address the inherent complexity and opacity of its mechanisms: explicability, understood as the requirement for AI systems to be both intelligible and explainable (Floridi, 2022, ch. 4). This principle, explicitly tailored to the governance of AI, is indispensable for ensuring that the other four principles can be effectively implemented (Floridi and Cowls, 2019). According to Floridi, then, explicability is an enabling principle in that it renders all other principles operational in the context of AI: it is «the crucial missing piece of the jigsaw when we seek to apply the framework of bioethics to the ethics of AI» (Floridi et al., 2018, p. 700).

Explicability thus assumes a dual significance: from an epistemological perspective, it concerns intelligibility, that is, the ability to answer the question “How does it work?”; while from a strictly moral perspective, it entails accountability, as it answers the question “Who is responsible for the way it works?”. (Floridi and Cowls, 2019; Floridi et al., 2018).

More specifically, the traditional principles of bioethics should be rearticulated to effectively respond to the emerging challenges introduced by AI. As indicated below:

(1) The principle of Beneficence requires that AI technologies be directed toward the good of humanity, placing the promotion of individual and planetary well-being at the center of their development and deployment;

(2) The principle of Non-Maleficence translates into a commitment to prevent harm arising both from human misuse or reckless application of AI, and from the behavior of inadequately designed systems;

(3) The principle of Autonomy calls for a careful balance between human decision-making power and that delegated to artificial agents, ensuring the protection of the intrinsic value of human choice in matters of significance;

(4) The principle of Justice demands that the development and use of AI generate equitably distributed benefits, while actively preventing the emergence of new forms of harm or discrimination;

(5) The principle of Explicability entails a sustained commitment to intelligibility and accountability. This means that we must be able to understand the effects of AI technologies on human society and the mechanisms by which they operate, while also ensuring that technology, and its human developers, can be held responsible for serious outcomes, through a traceable understanding of how such outcomes came about (Cowls and Floridi, 2018).

If explicability ultimately proves to be the essential principle for enabling the ethical use of AI (Amann et al., 2020), it simultaneously remains the punctum dolens of its application. The availability of an enormous volume of data and an increasingly complex network of operations, made possible by new technologies, comes at the cost of transparency, with the resulting impossibility of reconstructing the intricate web in which these countless relations are interwoven.

Without resigning ourselves to an opacity that inevitably compromises the capacity for critical assessment of outcomes generated by a tool which, precisely for this reason, demands constant ethical oversight, we must nevertheless begin to embrace a different perspective, one in which the human and technological worlds interact and increasingly blend into a continuous and uninterrupted flow of operations (Floridi, 2014).

## 3 Explicability, autonomy, informed consent: for a digital technology as support for medicine understood as a caring relationship

The adherence to the principle of explicability is thus indispensable for fostering the ethical deployment of AI across all sectors, ensuring transparency and intelligibility in AI decision-making for human users. Explainability, moreover, is essential for any medical procedure, and whatever technology is used, precisely because all medical procedures, in order to be conducted in substantial compliance with the autonomy principle, involve informed consent. In this regard, explainability could certainly be incorporated into the principle of autonomy, and it is not a notion that originated exclusively with the rise of AI. However, the opportunity to highlight its importance arises from the specific opacity introduced by AI decision-making processes. Building on this consideration, Floridi argues for the need to emphasize explicability to the extent of formulating it as an independent principle alongside the four principles of bioethics: more specifically, a principle with an enabling function in relation to the traditional principles of bioethics, aimed at strengthening the effectiveness of the existing framework in addressing the ethical implications entailed by the growing use of AI. Although a controversial hypothesis, it nonetheless provides a valid perspective for highlighting the critical issues introduced by the unprecedented opacity of AI-supported decisions, especially within the healthcare domain (Adams, 2023).

In the field of medicine, in particular, the need for explainability in algorithmic choices becomes especially pressing as AI increasingly permeates the patient-physician relationship, effectively becoming a third party within the most sensitive and constitutive phase of the therapeutic alliance, namely the process of informed consent. This moment represents a critical juncture, as the degree to which the patient’s choices can effectively align with the physician’s clinical recommendations depends on it.

In this regard, the capacity of patients to exercise autonomous choice, in accordance with the ethical principle of autonomy, is intrinsically linked to the efforts aimed at making the rationale underpinning AI-supported decisions both accessible and comprehensible. Consequently, explicability emerges as a necessary precondition for the effective realization of autonomy in the context of AI-assisted medicine. Absent such a guarantee, the principle of autonomy risks becoming devoid of substantive meaning, particularly when patients are expected to make consequential health-related decisions without adequate awareness of both AI’s influence on clinical judgment and the underlying logic of machine-driven processes.

Floridi’s notion of explicability is therefore intrinsically connected to the first of Beauchamp and Childress’s principles, autonomy, and to its principal expression: informed consent. As AI enters as a third party in the patient-physician relationship, it insinuates itself into the first and fundamental moment when the physician’s responsibility, precisely through informed consent, meets the patient’s autonomy.

A foundational pillar of both bioethics and biolaw, informed consent^3^ marks the shift from a paternalistic model of medical ethics to one grounded in the principle of autonomy. This paradigmatic turn is evident, beginning with the The Nuremberg Code (1947)^4^, in several key international documents: it is explicitly affirmed in the Declaration of Helsinki (1964, most recently updated in 2024) (World Medical Association, 2024)^5^, as well as in the Oviedo Convention on Human Rights and Biomedicine (Council of Europe, 1997)^6^.

It must be noted, however, that the formal recognition of this principle does not necessarily guarantee its substantive enforcement. While the ethical and legal importance of informed consent can no longer be seriously questioned, its implementation in everyday clinical practice is often undermined by bureaucratic drift, which tends to reduce it to a mere procedural formality (Allen et al., 2024).

Conversely, when considered in its full significance, informed consent reveals its complexity, as highlighted in the aforementioned Principles of Biomedical Ethics by Beauchamp and Childress. The classical text of principlism, within the section devoted to the principle of autonomy, outlines and examines the various critical issues surrounding informed consent.

In an extensive analysis, Beauchamp and Childress delineate the essential stages of informed consent. They begin by outlining its prerequisites (the competence, or the ability to understand and decide, and the voluntariness in deciding); they then distinguish between the informational components (disclosure of material information, recommendation of a plan, understanding of informations and recommendations), and the consent components (decision, in favor of a plan, authorization of the chosen plan) (Beauchamp and Childress, 2019, pp. 122 ff.). Additionally, a separate section addresses standard of surrogate decision making for non-autonomous patients (pp. 139 ff.).

What is of particular interest here is the section that examines, revealing its complexity, the process of communicating information to the patient. This appears to be the phase most affected by new and increasingly problematic elements, particularly due to the potential interference of AI, a factor not yet addressed in the text.

In general, as Beauchamp and Childress observe, the physician’s obligation to disclose information to the patient has been regarded as the most critical requirement of informed consent, the point at which the very rationale of the process is realized. Unsurprisingly, legal disputes over informed consent have often revolved around harms suffered by patients due to the intentional or negligent withholding of relevant information. Indeed, the very term “informed consent” first emerged within the legal context of such cases (Beauchamp and Childress, 2019, p. 123).

The key question to be resolved, today, concerns whether the introduction of AI may increase the risk of such omissions, thereby leading to a rise in legal disputes. In this regard, the communication of information concerning the use of AI is identified as a highly critical issue in the report to the Council of Europe: «Transparency and informed consent are key values in the AI mediated doctor-patient relationship. The complexity of AI raises a question: how should AI systems explain themselves, or be explained, to doctors and patients? […] AI systems interacting directly with patients should self-identify as an artificial system. Whether the usage of AI systems in care settings should always be disclosed to patients by clinicians and healthcare institutions is a more difficult question» (Mittelstadt, 2021, p. 5).

In what way, and within what limits, should the physician inform the patient about the use of AI?

To proceed rigorously in the consideration of this thorny question, it is certainly helpful to place it in the more general framework, already analyzed by Beauchamp and Childress, of the problematic nature of the information-communication process, starting with a question: does the physician have an obligation to inform the patient of everything he knows about his illness, including the diagnosis, possible treatment choices, and prognosis? Should he inform him of all risks, and all possible benefits, of all treatment alternatives? Or should it give him only some information, the information that is essential for him to make an informed choice whether to accept the proposed therapy or not? And again: is it possible to balance the completeness of information with the possibility of understanding on the part of the patient, initiating communication that leads to an informed choice? Or does such comprehensiveness make it difficult to understand, while, conversely, trying to make the information process understandable leads to oversimplification, with the risk of excluding some information?

Beauchamp and Childress (2019, pp. 124 ff.) propose standards of disclosure, pointing out that these standards are, not only legally, but also morally, relevant: (1) the professional practice standard; (2) the reasonable person standard; (3) the subjective standard.

The first standard holds that appropriate communication is determined by the customary practices of the medical profession: in short, it is up to the physician, who is assumed to always act for the good of the patient, to determine the amount and type of information to be given to the patient. Information communicated according to this standard, therefore, can only be challenged by other medical experts. Several critical issues have been highlighted in relation to the assumption of this standard, also referred to as “the reasonable doctor” standard. The first contention, today, appears of enormous importance: «It is uncertain in many situations whether a customary standard exists for the communication of information in medicine» (Beauchamp and Childress, 2019, p. 124).

The novelty of AI use, in fact, makes this uncertainty even more radical. Moreover, the standard of professional practice seems to be shifting the needle on the side of the physician, subverting the first intention that animates the practice of informed consent: to ensure the patient’s autonomous choice. And yet, paradoxically, it is perhaps the very complexity of the amount of information associated with the use of AI that brings this standard of professional practice back into play, when one thinks of the physician as a mediator between complex technical information and the patient’s ability to understand (Ferretti, 2025, pp. 107 ff.).

The second of the proposed standards bases the communication of information on what a “reasonable person” would consider important to know when deciding whether to undergo a particular treatment. While this standard undoubtedly has the merit of shifting the focus toward patient autonomy, it suffers from a lack of precision: it is not evident how to define a reasonable person, nor how to determine which pieces of information would be considered relevant. As a result, the standard remains largely theoretical and abstract, making it difficult to apply in practice (Beauchamp and Childress, 2019, pp. 124-125). When extended to a care relationship involving the patient, the physician, and AI, the challenge becomes even more pronounced: the complexity of the technology makes it harder to establish what a reasonable patient would be expected to want to know (Ferretti, 2025, p. 110).

The third standard moves away from the abstract assumption of an objective “reasonable patient” and instead grounds the extent of information in the specific and varying informational needs of each individual patient. Known as the subjective standard, this model is, from an ethical standpoint, the most desirable, as it is the only one that truly takes the patient’s autonomy seriously and considers their personal informational requirements. Nevertheless, this standard also presents practical challenges, both ethically and legally. Patients are not always aware of what they should be asking, nor can one expect the physician to have such intimate knowledge of each patient’s preferences as to know exactly what information they would wish to receive. Once again, a dual uncertainty emerges, one that becomes even more pronounced in the context of AI-assisted decision-making.

Despite this, the third standard opens a crucial path for addressing the challenges of communication, by turning attention to the individual subject. Beauchamp and Childress’ conclusion, in fact, is that we should start with the reasonable person standard, which we might interpret as an average measure of information, and then move on to try to address the subject’s specific information needs, articulated through a mutual exchange between patient and physician.

This conclusion appears to be consistent with what is stated in the Italian Medical Code of Ethics (FNOMCeO, 2025), specifically in Article 33 of the section Information and Communication with the Assisted Person, which affirms that it is the physician’s duty to ensure clear and comprehensive information. In the following sentence, the article also clarifies that «the physician adapt communication to the assisted person’s or their legal representative’s level of understanding, responding to all requests for clarification, and taking into account their emotional sensitivity and responsiveness».

Once again, the interference of AI, forcefully entering the patient-physician relationship as a third party, further complicates the already challenging goal of achieving communication in which the balance between completeness and comprehensibility is encapsulated in the notion of “adequacy”. The complexity of this new tool introduces additional barriers to understanding: some are subjective, stemming from the lack or insufficiency of technical knowledge on the part of both the physician and the patient; others are objective, some related to the inherent opacity, typical of machine learning systems, which process an enormous amount of data by following paths that in some ways elude the programmers themselves; others still are commercial in nature, resulting from the need for AI system manufacturers to protect trade secrets by withholding essential details about how their algorithms function (Zuddas, 2024, p. 594).

This gives rise to new and troubling questions: could these dual limitations lead the physician to relinquish the duty to inform, should it be deemed too burdensome, both for themselves and for the patient? Moreover, if the complexity of the new tool, verging on inexplicability, were so overwhelming as to prompt the patient to reject a treatment plan involving its use, could such a case of withheld information be legitimately framed within the so-called therapeutic privilege? This is the notion defined by Beauchamp and Childress as a privilege «which states that a physician may legitimately withhold information based on a sound medical judgment that divulging the information would potentially harm a depressed, emotionally drained, or unstable patient» (Beauchamp and Childress, 2019, p. 126).

In the case of uncertainty regarding whether to disclose information about the use of deep learning techniques, beyond the scenario involving a depressed, exhausted, or unstable patient, one might also reasonably encounter a patient who is anxious and struggles to maintain trust in the physician and the proposed treatment (De Menech, 2022, p. 196). In such cases, would it be inadvisable to inform the patient about the use of AI-based systems in the therapeutic process? The question is not an easy one to answer. While transparency remains a guiding principle, it may seem somewhat arbitrary to engage in detailed explanations of the inputs, outputs, and algorithms underpinning the specific system in use, particularly when neither the physician nor the patient possesses the technical expertise necessary to grasp such complexity.

Certainly, therapeutic privilege cannot either in this case, or in general, as Beauchamp and Childress make clear, be invoked on the basis that the disclosure of important information might lead the patient to refuse treatment (Beauchamp and Childress, 2019, p. 127). While it is reasonable for the physician to refrain from attempting a detailed explanation of the inputs, outputs, and algorithms on which the specific system is based, given the lack of sufficient technical competence on the part of both the physician and the patient, it is nonetheless appropriate for the physician to inform the patient that the system used in the therapeutic process is subject to human oversight. This ensures that its functioning is continually monitored and that any anomalies or unexpected consequences can be properly addressed (De Menech, 2022, p. 196).

All things considered, therapeutic privilege risks, in general, regressing the patient-physician relationship to a state prior to the institutionalization of the principle of informed consent, compromising the adequacy of communication that should characterize a genuine informational process by placing undue emphasis on the physician’s discretionary power.

## 4 Discussion

Ultimately, it is necessary to address the challenges currently faced by the process of informed consent, as the care relationship is no longer dyadic, between patient and physician, but triadic, involving the patient, the physician, and AI. In this new context, it remains essential to uphold the key foundational values of this relationship: informed consent and transparency (Zuddas, 2024, p. 596; Mittelstadt, 2021, p. 5).

Therefore, the real issue at the heart of this debate should not be whether to inform patients, but rather how to inform them (Zuddas, 2024, p. 594).

With regard to the limitations encountered in the complex process of information and communication that lies at the core of the care relationship, if we first examine the subjective limitations, namely, those arising from the limited knowledge of both physician and patient, two possible approaches may be considered to overcome them. The first, and most obvious, solution lies in simplifying the technical information (Chau et al., 2025), reducing it to its essential elements. The physician’s duty to inform the patient about the use of AI can be considered fulfilled if the physician limits themselves to explaining in general terms how the technology employed works, presenting its typical benefits and limitations (De Menech, 2022, p. 195).

Such simplification of information may benefit both physician and patient by facilitating communication; however, it presupposes a solid foundation in the subject matter in order to avoid the risk of omitting essential data or overlooking important questions. This reflects the difficult but necessary effort to overcome the subjective limitation described in the joint report by the Italian National Bioethics Committee (CNB) and the National Committee for Biosecurity, Biotechnology, and Life Sciences (CNBB), which calls for greater investment in education, not only for physicians (Weidener and Fischer, 2024), but also for the general public, in the field of computer science: the aim is promoting broader awareness of the various forms of AI and their applications, particularly in medicine (CNB - CNBBVS, 2020, pp. 15 ff.).

In a working environment increasingly shaped by AI assistance (Borghi et al., 2025), it is also essential to invest in the continuous development of physicians’ soft skills (Lu et al., 2024).

Even more challenging is the task of overcoming the objective limitations that affect the information process due to the interference of AI. These limitations stem from the problem of structural opacity inherent in automated processes, that often follow such complex internal logics as to render them incomprehensible to the human mind. Therefore, the physician may find themselves in the pressing situation of having to communicate information about AI processes that remain opaque even to the system’s own developers.

With regard to this second and more serious concern, we might, for the time being, echo what has already been said in the field of law, something that applies even more fundamentally to ethics: it is essential not to merely chase after AI applications, but rather to act upstream, by establishing principles and rules “by design”, from the outset (Casonato, 2019, p. 725).

The fifth principle that Floridi adds to the four proposed by Beauchamp and Childress, explicability, highlights precisely the need for AI systems to be explainable and intelligible.

At the intersection of ethics and law, informed consent, central to the patient-physician relationship, must retain its role even when the introduction of AI inserts a third party between the two original interlocutors. This centrality implies that anything which cannot be the object of comprehensible communication cannot, in turn, be designed as the exclusive “tool” for practices such as medicine, practices that seek to uphold and honor human dignity.

Hence, to conclude by drawing on the words of Mittelstadt, «the doctor-patient relationship is a keystone of “good” medical practice, and yet it is seemingly being transformed into a doctor-patient-AI relationship. The challenge facing AI providers, regulators, and policymakers is to set robust standards and requirements for this new type of “healing relationship” to ensure patients’ interests and the moral integrity of medicine as a profession are not fundamentally damaged by the introduction of AI» (Mittelstadt, 2021, p. 7).

Emerging technologies can indeed contribute to increased efficiency in various fields, provided they are deployed within a normative framework grounded in values and moral principles that are compatible with a pluralistic society. Crucially, this calls for the clear identification, critical recognition, and deliberate safeguarding of those domains of human labor and activity that cannot be substituted by machines.

## Footnotes

1 In this context, the expression patient-physician is preferred over the more conventional physician-patient in order to emphasize the logical and ethical primacy of the patient within the therapeutic relationship. This reversal also appears in the aforementioned opinion issued by the CNB and CNBBVS on artificial intelligence and medicine.

2 As established by Italian law, «the time dedicated to communication between physician and patient shall be considered time of care». Law no. 219 of December 22, 2017, Article 1, paragraph 8). [https://www.gazzettaufficiale.it/eli/id/2018/1/16/18G00006/sg]

3 In Italy, informed consent and its reverse, informed dissent, are the subject of law 219 of 2017, Rules on informed consent and advance processing provisions.

4 The Nuremberg Code (1947), British Medical Journal No 7070 Volume 313: page 1448, 7 December 1996chromeextension://efaidnbmnnnibpcajpcglclefindmkaj/https://media.tghn.org/medialibrary/2011/04/BMJ_No_7070_Volume_313_The_Nuremberg_Code.pdf

5 WORLD MEDICAL ASSOCIATION, Declaration of Helsinki 2024, Medical Research Involving Human Participants, https://www.wma.net/what-we-do/medical-ethics/declaration-of-helsinki/

6 COUNCIL OF EUROPE, Convention for the Protection of Human Rights and Dignity of the Human Being with regard to the Application of Biology and Medicine: Convention on Human Rights and Biomedicine, Oviedo,4.IV.1997, https://www.coe.int/en/web/conventions/full-list?module=treaty-detail&treatynum=164
